# Global warming-related response after bacterial challenge in *Astroides calycularis*, a Mediterranean thermophilic coral

**DOI:** 10.1038/s41598-024-58652-0

**Published:** 2024-04-11

**Authors:** L. Bisanti, C. La Corte, M. Dara, F. Bertini, M. G. Parisi, R. Chemello, M. Cammarata, D. Parrinello

**Affiliations:** 1 Department of Earth and Marine Sciences, University of Palermo, 90128 Palermo, Italy; 2 NBFC, National Biodiversity Future Center, 90133 Palermo, Italy

**Keywords:** Ecology, Physiology, Climate sciences, Environmental sciences

## Abstract

A worldwide increase in the prevalence of coral diseases and mortality has been linked to ocean warming due to changes in coral-associated bacterial communities, pathogen virulence, and immune system function. In the Mediterranean basin, the worrying upward temperature trend has already caused recurrent mass mortality events in recent decades. To evaluate how elevated seawater temperatures affect the immune response of a thermophilic coral species, colonies of *Astroides calycularis* were exposed to environmental (23 °C) or elevated (28 °C) temperatures, and subsequently challenged with bacterial lipopolysaccharides (LPS). Using immunolabeling with specific antibodies, we detected the production of Toll-like receptor 4 (TLR4) and nuclear factor kappa B (NF-kB), molecules involved in coral immune responses, and heat shock protein 70 (HSP70) activity, involved in general responses to thermal stress. A histological approach allowed us to characterize the tissue sites of activation (epithelium and/or gastroderm) under different experimental conditions. The activity patterns of the examined markers after 6 h of LPS stimulation revealed an up-modulation at environmental temperature. Under warmer conditions plus LPS-challenge, TLR4-NF-kB activation was almost completely suppressed, while constituent elevated values were recorded under thermal stress only. An HSP70 up-regulation appeared in both treatments at elevated temperature, with a significantly higher activation in LPS-challenge colonies. Such an approach is useful for further understanding the molecular pathogen-defense mechanisms in corals in order to disentangle the complex interactive effects on the health of these ecologically relevant organisms related to global climate change.

## Introduction

Direct and indirect impacts of global warming are proving detrimental effects to the health of marine species and are the primary cause of coral death worldwide^1–3^, as well as affecting the Mediterranean^4,5^. The coral holobiont is an obligate association between the coral animal and a plethora of mutualists (i.e., an appropriate commensal microbiota), including dinoflagellate endosymbionts of zooxanthellate species^6^; however, this association is delicately balanced, and coral species worldwide are increasingly threatened by warming seawater and temperature-driven disease outbreaks^1,7–9^. Indeed, the risk of climate anomalies in the Mediterranean basin has increased sharply over the last few decades, with warming temperatures exceeding the range of normal fluctuations historically experienced by marine organisms^5,10,11^. This trend has boosted well-documented disease outbreaks and, consequently, mortality events that have affected several anthozoan species across varying geographic extents^4,12–14^. Despite recent advances, much remains to be understood about the molecular processes that underpin coral immune responses to pathogens. Consequently, it is crucial to determine the immune mechanisms involved and how they may be influenced by environmental signals (e.g., temperature).

From an anatomical point of view, corals have a simple body structure consisting of only two true layers of tissue, and no organs. Depending on the stage of development – developing polyp or adult coral—these tissues are called ectoderm/epithelium and endoderm/gastroderm, respectively, interlined by the mesoglea (an amorphous and practically acellular extracellular matrix)^15,16^. In zooxanthellate species, *Symbiodinium* spp. (coral-dinoflagellate symbiont) reside mainly in the gastrodermal areas between the gut and the external epithelium (barrier to the surrounding environment) in the oral end, and more rarely in the gastrodermal areas between the intestine and the skeleton of the organism. Instead, nematocysts are present only in the epithelium of the oral end, but not in the calicoblastic epithelium^16^. The majority of host corals have only species-specific associations with specific bacterial phylotypes (including *Symbiodinium* spp.) which populate the different habitats of coral anatomical compartments, such as the surface mucus, tissues, and skeleton^17,18^. This suggests the presence of a well-developed self/non-self-recognition system in which appropriate strains multiply by establishing a stable symbiosis, while unsuitable strains are actively removed through cellular processes^6,19,20^.

Corals and other cnidarians contain a surprisingly high degree of genetic complexity^21–24^, with homologs of many proteins involved in vertebrate immunity being described for these organisms in the last two decades^16,23,25,26^. The primary function of the anthozoan innate immune system is to recognize specific patterns of non-self-entities^27,28^. These patterns are called pathogen-associated molecular patterns (PAMPs), or microbe-associated molecular patterns (MAMPs), such as lipopolysaccharide (LPS), peptidoglycan, and mannan components of the microbial cell wall. These and other PAMPs are recognized by proteins called pattern recognition receptors (PRRs), which are molecules that include both membrane-bound proteins, such as Toll-like receptors (TLRs), and soluble proteins, such as lectins^1,16,25,29^. Corals possess TLRs and nucleotide oligomerization domain (NOD)-like receptors (NLRs) with intracellular Toll/interleukin-1 receptor (TIR) domains which can interact with genetic homologs of myeloid differentiation primary response protein 88 (MyD88), IL-1R-associated kinase (IRAK), receptor-associated factor 6 of TNF (TRAF), and IkB kinase (Ikk), which cleave nuclear factor kappa B (NF-kB) inhibitors and allow NF-kB protein dimers to translocate into the nucleus, enhancing the expression of inflammatory cytokines, the production of antimicrobial peptides (AMPs), cell survival, and apoptosis^16,25,29,30^. For example, the innate immune pathway from TLR to NF-kB in the tropical species *Orbicella faveolata* was recently characterized. Compared to human TLRs, the intracellular TIR domain is very similar to TLR4, and treatment of *O. faveolata* tissue with lipopolysaccharides (LPS; a common ligand for mammalian TLR4) led to changes in gene expression consistent with the mobilization of the NF-kB pathway^26^.

One group of proteins used as ubiquitous and putative markers of temperature-induced cellular stress in corals are the heat shock proteins (HSPs)^31–35^. HSPs are differentiated by molecular weight into several major chaperone families (HSP40, HSP60, HSP70, HSP90, HSP100, and the small HSPs), with specific intracellular localization and function^36^. As molecular chaperones, they support protein homeostasis by facilitating proper protein folding and translocation, and by aiding in the folding or degradation of proteins damaged by heat or other environmental stresses^37,38^. The involvement of these proteins in the immune system has been widely reported in both vertebrates and invertebrates, increasing after exposure to biotic challenges such us during the development of infections and/or diseases^39,40^. For example, human HSP70 activates the TIR receptor signaling pathway during a highly inflammatory response^39,40^. For corals, the increased genetic expression of HSP70 in tropical species colonies showing signs of disease indicates their potential involvement in the immune and stress responses to pathogenic challenges^41,42^.

The present study, using a manipulative aquarium-based experiment, aims to assess the impacts of warmer conditions on the activity of TLR4, NF-kB, and HSP70 markers under bacterial LPS challenge in an azooxanthellate coral species, *Astroides calycularis* (Pallas, 1766). This species is commonly found in the central-southern part of the Mediterranean Sea, covering vertical rocky reefs, overhangs, and caves below the intertidal fringe^43^. *A. calycularis* occupies both well-lit and sciaphilous habitats and is considered a tolerant thermophilic species, thriving at relatively high temperatures^43^. However, recent field observations indicate that this orange coral is impacted by seawater temperatures reaching and exceeding 28 °C^44,45^. In this experiment, the use of bacterial LPS allowed us to avoid the well-documented difficulties of infecting coral with live strains^46^, while testing the induction/suppression of immune pathways by PRRs^15,47,48^.

## Results

### Western blot analysis and cross reactivity validation

**Figure 1:**
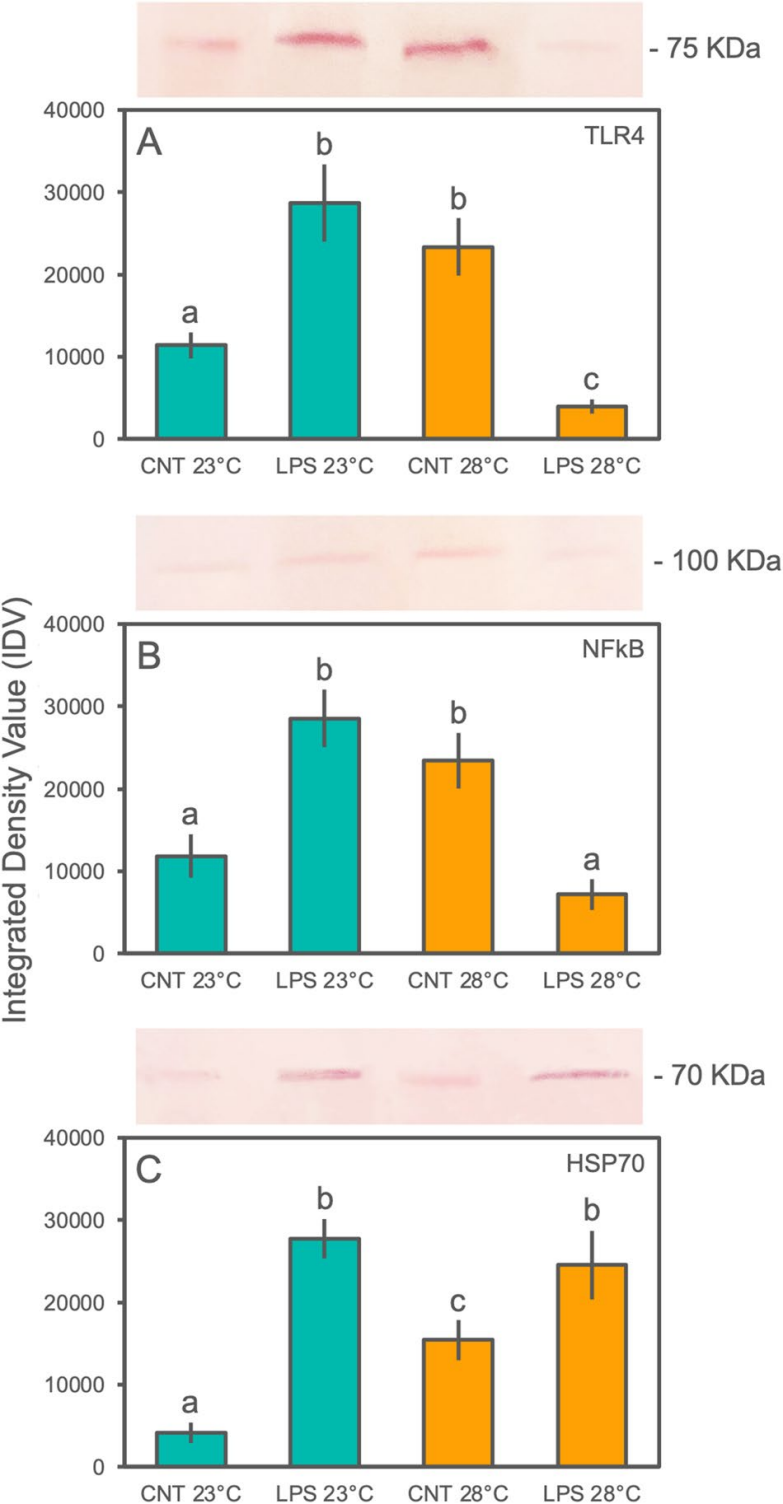
Western blot representative assay carried out on *A. calycularis* tissue extracts from the experimental treatments. Each nitrocellulose sheet was treated with anti-TLR4, anti-NF-kB, and anti-HSP70, the anti-rabbit IgG-alkaline phosphatase or anti-mouse IgG-alkaline phosphatase secondary antibody, and developed with the BCIP/NBT liquid substrate system (see Supplementary material for full-length blotting images). Plot indicates the relative Integrated Density Value (IDV; mean values ± SD) calculated on bands of the Western blot assays. The letters indicate statistically significant differences (*p* < 0.05) between experimental groups.

Western blot assays were performed on protein extracts of *Astroides calycularis* confirming the presence in sampled tissues of TLR4, NF-kB, and HSP70-immunoreactive bands. The analysis carried out with the anti-TLR4, anti-NF-kB, and anti-HSP70 antibodies revealed bands of approximatively 75, 100, and 70 kDa, respectively, corresponding to the expected molecular weights of these proteins (Fig. S1, S2, S3). The expression of TLR4, NF-kB, and HSP70 was investigated among the different experimental treatments (Fig. 1). In detail, the activity of TLR4 differed significantly among treatments (Fig. 1A; Table 1). At environmental seawater temperature, higher densitometry evaluation of the bands by integrated density values (IDV) were found for the LPS-challenge group of corals compared to unchallenged specimens. However, *A. calycularis* demonstrated a significant change in expression driven by warmer temperature alone, with an increase in IDV comparable to the LPS-challenge group at environmental temperature, while, a significant inhibition was recorded for animals under LPS-challenge and elevated temperature. A similar trend was shown for the NF-kB activity, with significantly higher IDV values for colonies which were LPS-challenged at environmental temperature and LPS-unchallenged at warmer seawater conditions, compared to the other treatments (Fig. 1B; Table 1). Significant differences were found for HSP70 activity (Fig. 1C; Table 1). Under controlled seawater temperature (23 °C), a significant up-regulation was recorded for LPS-challenged specimens compared to control colonies. For the elevated temperature condition, a significant and positive modulation in LPS-unchallenged samples was found compared to environmental controls, though it was lower than the specimens exposed to LPS; effectively, the LPS-challenged corals showed significantly higher IDV values compared to controls (both under environmental and elevated temperature conditions), as well as to the LPS-challenged specimens under environmental conditions.

**Table 1:** Summary of the one-way ANOVAs carried out among experimental treatments on integrated density values of the investigated markers (TLR4, NF-kB, and HSP70) in the orange coral *A. calycularis*.

One-way ANOVA	*F*	*P* value	*R* square
TLR4	66.71	< 0.0001	0.92
NF-kB	57.73	< 0.0001	0.91
HSP70	73.07	< 0.0001	0.93

### Histological analysis and immunohistochemistry

**Figure 2:**
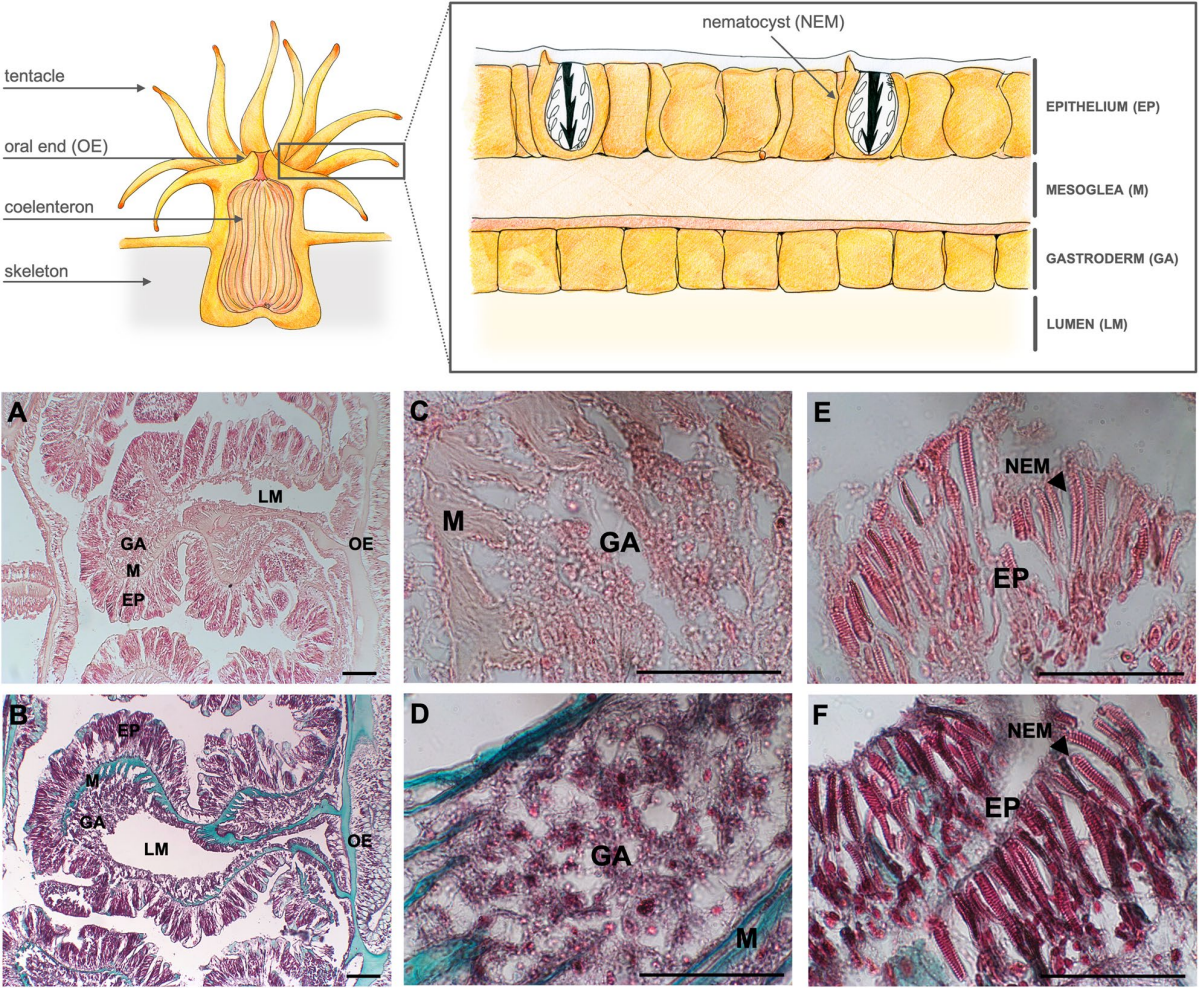
Schematic anatomical representation and histological sections of adult *A. calycularis* corals showing the different tissues of the gastroderm (GA), mesoglea (M), epithelia (EP) and the specific structures of nematocysts (NEM) at the oral end. Coral sections are shown after staining with: (**A**–**C**–**E**) hematoxylin/eosin and (**B**–**D**–**F**) Gomori trichrome, to act as a reference for structural detail. Scale bar 50 μm.

Generally, tissues of *A. calycularis* were characterized by an epithelium formed by a large cellular monolayer arranged obliquely with the basal part lying on the mesoglea (Fig. 2A, B), populated by nematocysts even in the innermost portion at the oral end (Fig. 2E, F). No nematocysts were present in the calicoblastic epithelium toward the aboral end. The mesoglea was clearly visible between the epithelium and the gastroderm, consisting of an amorphous fibrous matrix (Fig. 2A, B). Instead, the underlying gastrodermal area of the orange coral was made up of a single, thinner layer, but characterized by smaller cells densely packed together (Fig. 2C, D).

**Figure 3:**
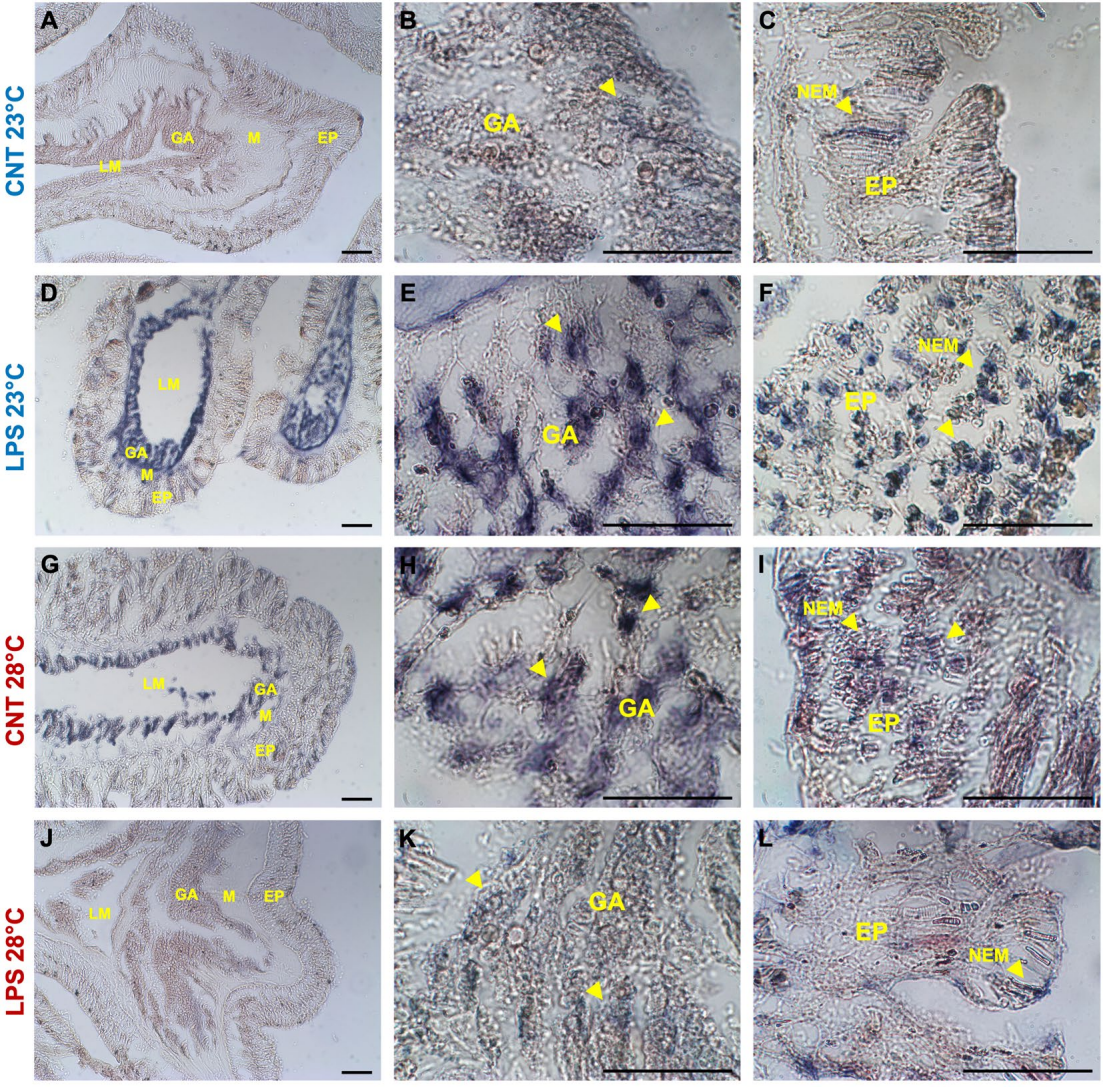
Immunohistochemical detection of TLR4 in oral end tissues of adult *A. calycularis* corals for the experimental treatments, with the primary antisera anti-TLR4. Yellow arrows indicate immunostaining in tissues or specific structures. Gastroderm (GA); mesoglea (M), epithelia (EP); nematocysts (NEM). Scale bar 50 μm.

**Figure 4:**
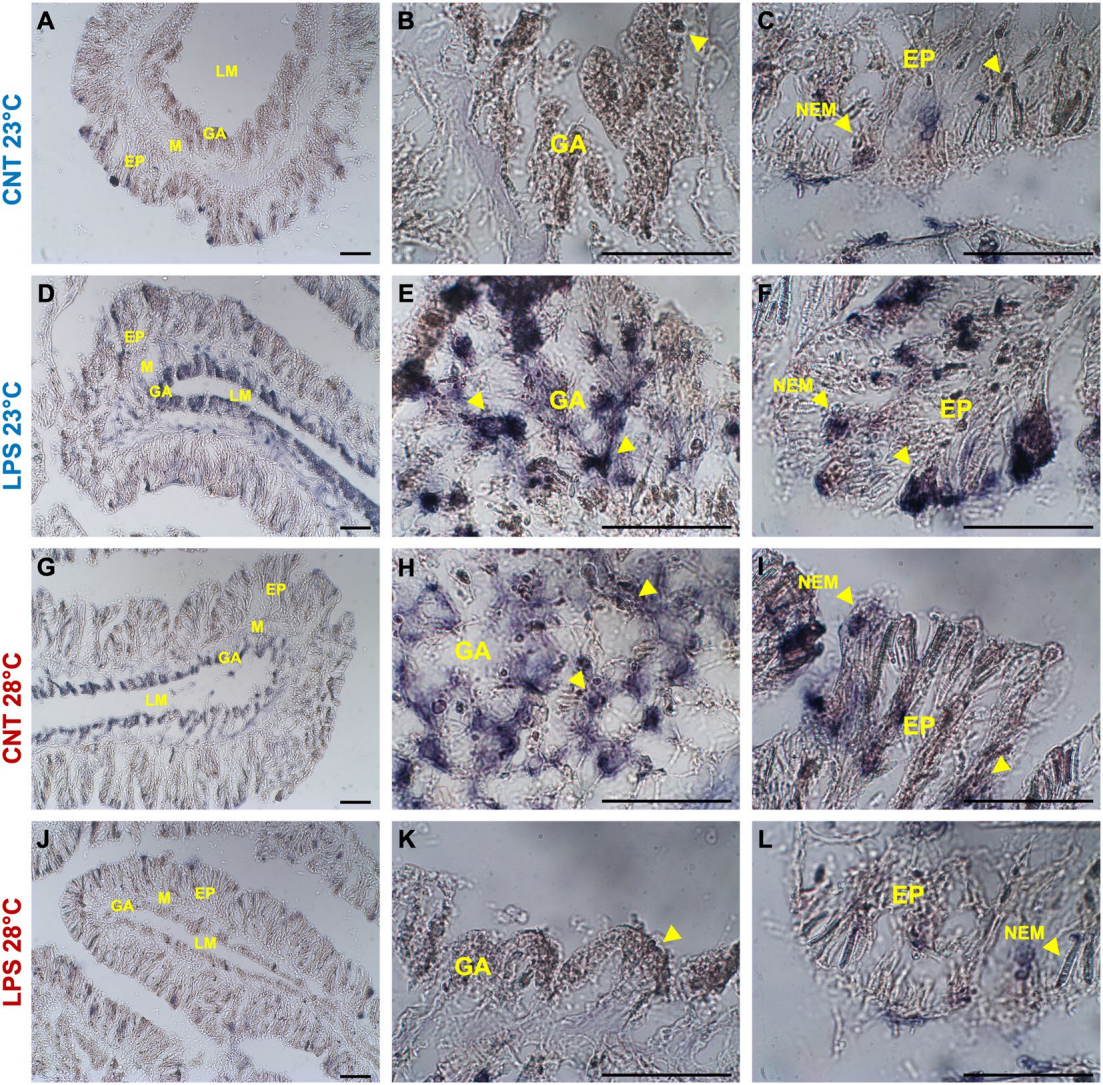
Immunohistochemical detection of NF-kB in oral end tissues of adult *A. calycularis* corals for the experimental treatments, with the primary antisera anti-NF-kB. Yellow arrows indicate immunostaining in tissues or specific structures. Gastroderm (GA); mesoglea (M), epithelia (EP); nematocysts (NEM). Scale bar 50 μm.

**Figure 5:**
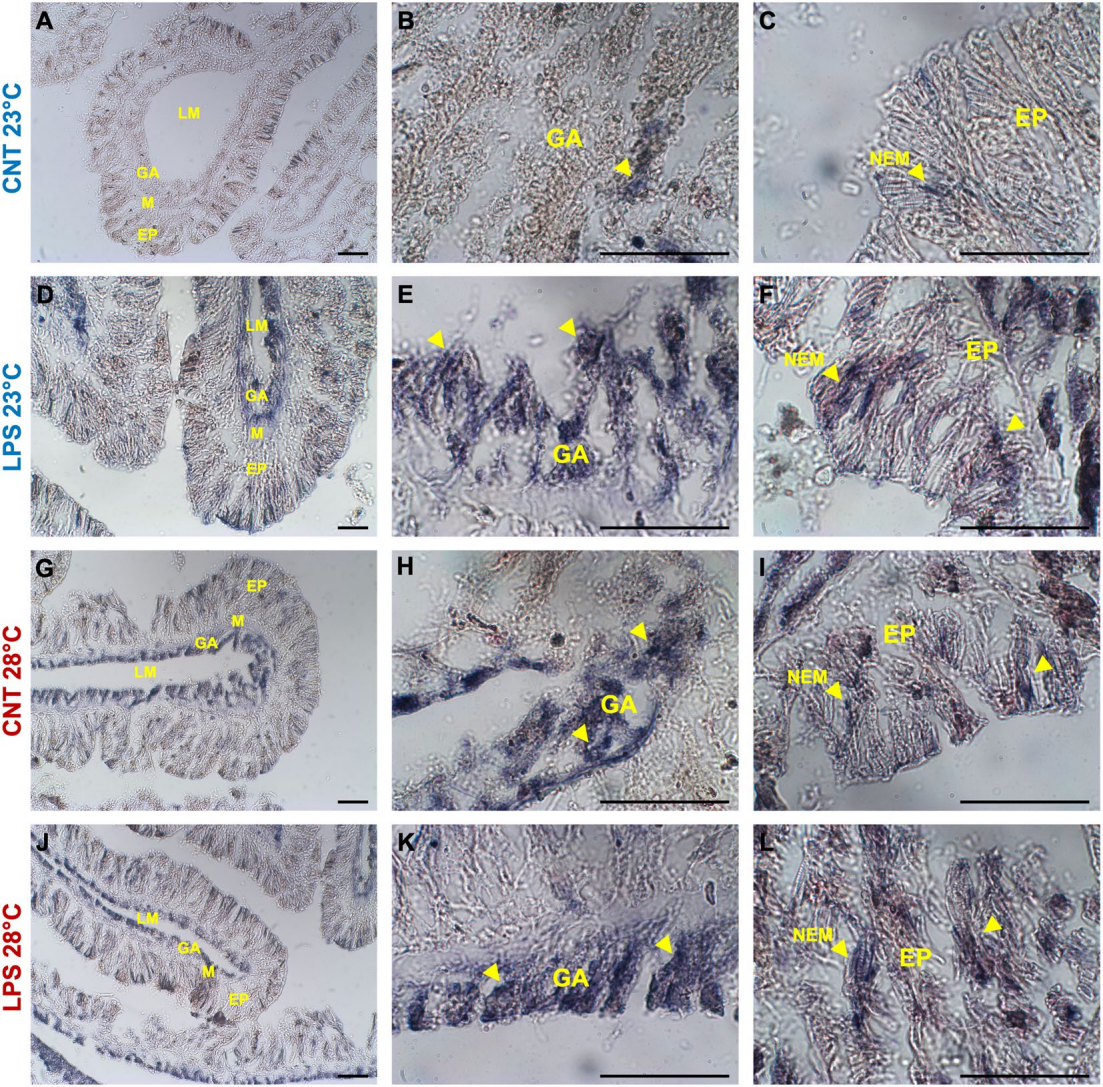
Immunohistochemical detection of HSP70 in oral end tissues of adult *A. calycularis* corals for the experimental treatments, with the primary antisera anti-HSP70. Yellow arrows indicate immunostaining in tissues or specific structures. Gastroderm (GA); mesoglea (M), epithelia (EP); nematocysts (NEM). Scale bar 50 μm.

**Figure 6:**
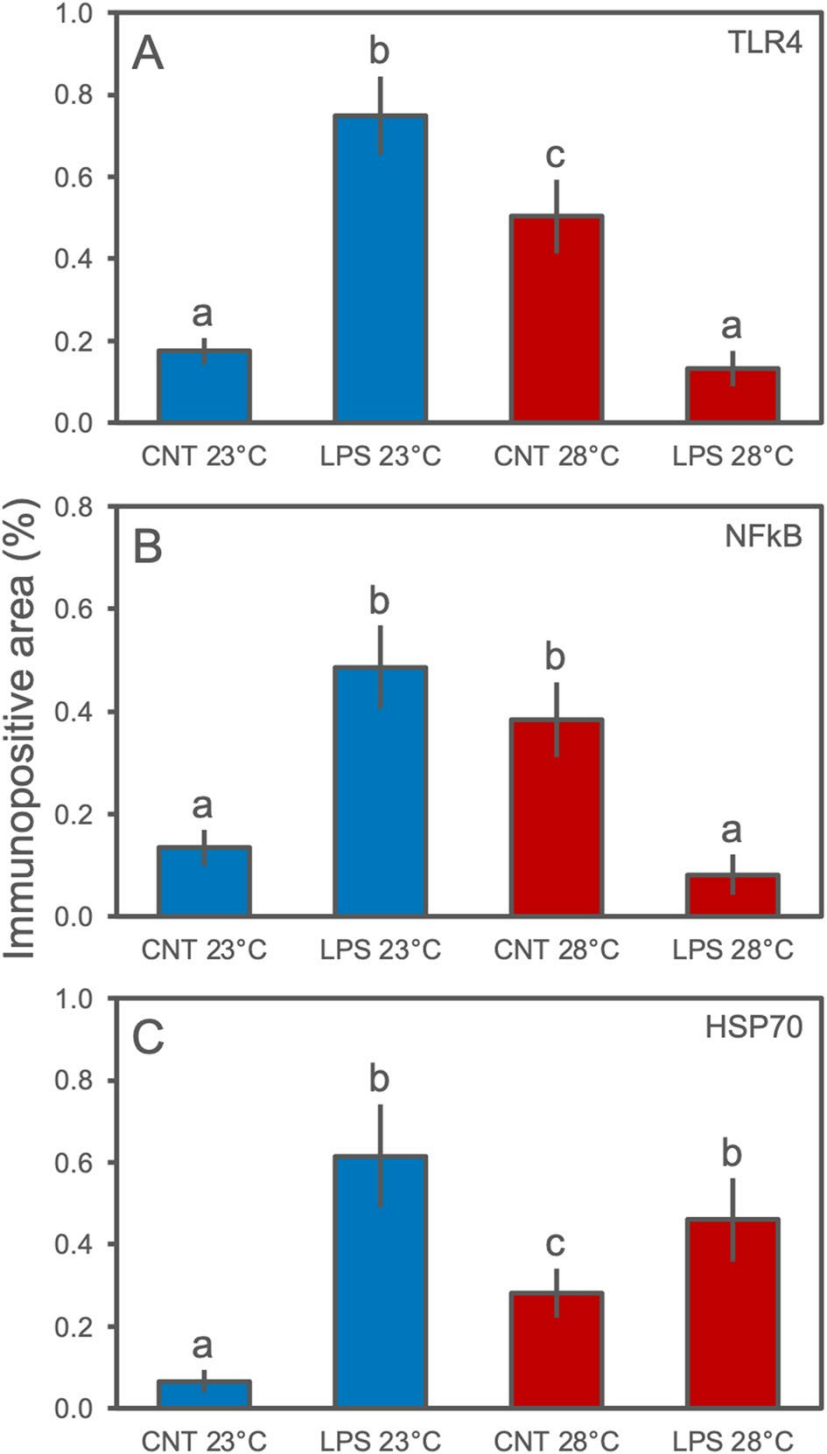
Quantification of the immune-positive stained areas (percentage of stained cells; mean values ± SD) in *A. calycularis* tissues from slides of the experimental treatments. The analysis was carried out considering all tissue districts of the orange corals (i.e., from oral to aboral ends). The letters indicate statistically significant differences (*p* < 0.05) between experimental groups.

The immunohistochemistry assays performed on sections of LPS-challenged corals showed a strong signal for TLR4, NF-kB, and HSP70 at environmental temperature, compared to control slides, in both gastrodermal cells as well as in the nematocysts of the epithelium at the oral end (Figs. 3, 4, 5A–F). Immunostaining was also observed in some compartments of the gastrodermal lining of the gut and skeleton (photos not shown). No staining was observed in pre-immune serum controls for either of the primary antibodies or when the primary antibody was omitted. The quantification of the percentage of the immune-positive areas indicated significant differences in the immunostaining for the three markers considered, with higher values for the LPS-challenge treatment compared to control (Fig. 6; Table 2). By contrast, under warmer seawater conditions, the amount of TLR4 and NF-kB immunostaining signals of antibody recognition unveiled that they were constitutively over-expressed in LPS-unchallenged coral tissues (Figs. 3, 4G–L). Staining patterns were similar to the slides of samples exposed to LPS at 23 °C, with an extended signal associated with nematocysts in the coral epithelium and gastrodermal cells (Figs. 3, 4G–I). Low immune-positivity was found in tissues of LPS-challenged polyps for both markers (TLR4 and NF-kB) (Figs. 3, 4J–L). Also in this case, the analysis of the percentage of immune-positive areas showed statistically significant differences between experimental treatments (Fig. 6A, B; Table 2).

**Table 2:** Summary of the one-way ANOVAs carried out among experimental treatments on immune-positive stained areas (percentage values) of the investigated markers (TLR4, NF-kB, and HSP70) in the orange coral *A. calycularis*.

One-way ANOVA	*F*	*P* value	*R* square
TLR4	86.15	< 0.0001	0.94
NF-kB	51.05	< 0.0001	0.90
HSP70	36.82	< 0.0001	0.87

On the other hand, at elevated temperature, the HSP70 marker showed a different staining pattern in the orange coral tissues. A clear immuno-positive signal for both treatments (control and LPS-challenged) was detected (Fig. 5G–L), although the analysis of the percentage areas of stain-cells showed significantly higher values for the samples exposed to LPS (Fig. 6C; Table 2).

## Discussion

The present study investigated the effects of warmer seawater conditions and LPS-challenge (simulating a potential pathogen aggression) on the expression of selected immunological (TLR4 and NF-kB) and stress (HSP70) markers in the Mediterranean scleractinian species *A. calycularis*. Both these types of abiotic and biotic factors are recognized as being among the most important contributors to the worldwide decline of coral habitats and mass mortality events^4,5,7,8,49^. Indeed, in recent decades, efforts to understand the cellular processes involved in the immunological and physiological responses to environmental stresses that cause mortality have increased. This is significant, especially considering that these animals cannot move to new optimal environmental conditions and that the earliest steps of the organism’s response occur at the cellular level^50,51^.

To date, the regulation of the TLR-NF-kB pathway performed in corals under bacterial challenge has confirmed that these organisms possess temporally dynamic and responsive cellular machinery to counteract stresses^26,52–54^. Consistently with this, the results of our experiment showed significant activity modulation in corals at environmental temperature, for both TLR4 and NF-kB markers. Immunostaining using specific antibodies on sections of coral colonies exposed to LPS resulted in strong staining of nematocysts in the epithelia compared to control. Dense cellular aggregates lining the gastroderm directly adjacent to the lining of the gut in both oral (facing the mouth and external environment) and aboral (lining the skeleton) tissue were also stained. This wide activation is coherent with the expression levels in LPS-challenged samples detected by the Western blot analysis in the present study. Localization of the TLRs to both the nematocysts and the gastroderm would ensure that pathogens are exposed to receptor proteins regardless of how they enter the corals, thus activating the organism’s immune response. Given the binding capabilities of bacterial cell walls by TLRs, this exposure could guarantee an effective means to inhibit further tissue colonization. The activation times observed in this study (i.e., after 6 h of LPS exposure) are also consistent with previous observations, which showed up-regulated levels of mRNA encoding several TLR-NF-kB pathway components approximately 4 h after treatment of a tropical coral species with LPS^26^. Under healthy control conditions, *A. calycularis* therefore appears to possess the resources necessary to activate the TLR-NF-kB pathway after 6 h of LPS exposure. These immune pathways are essential in coral response to disease, and generally their tight regulation is a balanced consequence of signaling, organismal conditions, and overall immune strategy^55^.

Under elevated temperature conditions, a significant up-regulation in TLR4 and NF-kB markers of LPS-unchallenged specimens was shown. Increased expression of several components involved in the TLR-NF-kB pathway has also been described in several anthozoans following heat stress treatment^56–59^. This provides further evidence that the innate immune system of corals is sensitive to environmental changes^48,60,61^. Conversely, when exposed to LPS challenge and warmer seawater conditions simultaneously, a significant suppression in both immune markers’ activities was observed, with a lack of extensive immunostaining in both the epithelial and gastrodermal tissues of the corals. Since the TLR-NF-kB signaling pathway also regulates AMP expression, the orange coral may reduce the production of these key molecules and, therefore, be unable to respond effectively to disease under thermal stress. Although no AMPs have yet been identified in *A. calycularis*, genes encoding AMPs have been characterized for several scleractinian corals and in other cnidarians^62–66^. Indeed, AMPs have been shown to be crucial in actively regulating and maintaining the health of the tissue-associated bacterial community in several species of anthozoans^62,67,68^. The suppression of these immuno-dynamics could have serious implications for this Mediterranean species during the summer months, when seawater temperatures are higher and the risk of disease outbreaks increases in the basin^4,12–14^. Despite these considerations, a limitation of this work is that it only considers one-time post-exposure to LPS, providing partial information about the organism's responses. Understanding and/or identifying the relevant timing of immune activities will be crucial for avoiding underestimates of the response capability to stress, and therefore the survival, of these ecologically relevant organisms.

With regard to HSP70, a significant up-regulation was detected in LPS-challenged corals at environmental temperatures. Compared with the control slides, the immunohistochemical analysis showed an effective, wide activation in all tissue districts of the organisms. These findings suggest that changes in HSP70 occur in corals in response to microbial aggressions, also in the absence of thermal stress, further supporting their role in the coral immune response^34,42^. Indeed, several studies conducted on invertebrates (including corals) have already shown that modulations of HSP70 production appear to protect the organism from pathogenic infection^34,40,42,69^. For example, evidence that HSP70 enhances resistance to pathogens by priming and enhancing the expression of the pro-phenoloxidase system has already been presented^69^. Extracellular HSPs could also represent the ancestral danger signal of cell death or lysis-activating innate immunity^70^. In the present study, an increase in HSP70 protein production following LPS recognition could be an attempt to protect the organism, possibly by activating other constitutive components of the coral effector immune systems (e.g., via activation of the pro-phenoloxidase cascade).

Another novel result from this study is a significant modulation of HSP70 activity at elevated seawater temperature, for both control and LPS-challenged treatments. The unchallenged corals showed significantly higher values than the controls at environmental temperature, though not reaching those of LPS-challenged colonies (both at environmental and elevated temperature). Indeed, under thermal stress, the key role of cytoplasmic HSPs (including HSP70) in several cellular processes has already been widely demonstrated; these include the folding of newly synthesized and misfolded proteins, the stabilization of the cytoskeleton, and protein transfer to other cellular compartments^38,71^. When LPS-challenged, *A. calycularis* showed a significantly up-modulation than control corals, also occurred in terms of increased areal activation of immunostaining tissues. The emerging dynamics suggest that, under warmer seawater conditions, the organism is able to activate a moderate response to thermal stress alone, probably in order to maintain homeostasis; instead, corals stimulated by LPS are able to further implement their response through the sensitive regulation of HSP70 production similar to that at environmental temperature. However, to confirm such immune dynamics under warmer conditions and pathogen eliciting in corals, studies over longer time intervals and with higher sampling resolution would be required.

In conclusion, these results demonstrate that the immune response of *A. calycularis* exhibited 6 h post LPS-challenge is significantly affected by warmer seawater conditions. While thermal stress alone stimulated the activation of the coral TLR-NF-kB pathway, responses to LPS under elevated temperature were almost completely suppressed. HSP70 activity was up-modulated for both treatments under thermal stress, with the response of LPS-challenge colonies significantly stronger compared to control corals. These non-linear, temperature-induced responses of the examined markers could be the result of energetic trade-offs between maintaining homeostasis and the costs incurred to implement an effective immune response by the organism, occurring within the predetermined constraints of evolution^55,72,73^. Such an immuno-ecological approach represents a challenging path for evaluating immunocompetence and understanding natural patterns of disease among coral species across habitats. This study provides new biological information on an endemic Mediterranean species that can be used to better understand ecological patterns and, therefore, increase the accuracy of predicted responses to future climate-related events.

## Materials and methods

### Coral collection and experimental design

**Figure 7:**
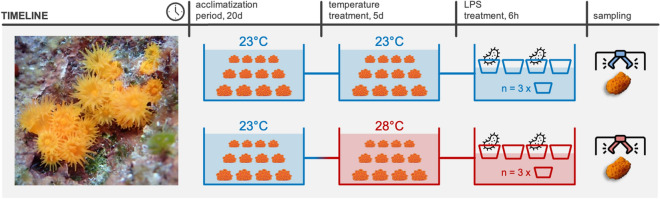
Schematic drawing summarizing the experimental timeline. Experimental treatments were carried out in duplicate.

As described in Fig. 7, 54 comparably sized (3.5 ± 1.0 cm colony diameter) and visibly healthy adult colonies of *Astroides calycularis* were sampled in October 2022 from the upper littoral zone (~ 4–5 m depth) of Capo Zafferano Bay (38° 11′ 11″ N, 13° 53′ 82″ E; NW coast of Sicily, Italy). Each colony was carefully removed with the aid of a hammer and chisel and transported in a 1 µm filtered seawater tank to the laboratory. At the Marine Immunobiology Laboratory (Department of Earth and Marine Sciences, University of Palermo), corals were equally and randomly assigned among four large aquaria supplied with continuous flow-filtered seawater (1 µm) at environmental temperature during the sampling periods (daily averages temperature ranged from 22.90 ± 0.17 to 23.54 ± 0.01 °C). Aquaria conditions were maintained with a 15:9 h photoperiod of daily light/dark cycles to match the natural photoperiod at the collection sites (metal halide lights, with levels maintained at 150–250 µmol m^-2^ s^-1^), and in dimmed light conditions to mimic their natural sciaphilous environment (the tanks were under a canopy of 70% light-reducing shade-cloth). During the 20-day acclimatization period, the colonies were fed twice a week with a commercial preparation of plankton (Elos Coral Foods SvC) prior to experimentation^74^. After the acclimatization period, 3 randomly selected samples were fixed for the histological experiments (detailed in the histological analysis section) or snap frozen and stored at -30 °C for tissue extraction (detailed in the Western blotting section) as pre-treatments controls. Two aquaria were then randomly selected for the elevated temperature treatment, in which temperature was increased by 1.0 to 1.5 °C per day for 3 days and stabilized at 28 °C (daily averages temperature ranged from 28.00 ± 0.17 to 28.41 ± 0.19 °C).

Two small plastic tanks, holding ~ 5 l seawater and equipped with individual air-stones, were submerged within each of the four large aquaria, for a total of 8 small tanks. Three colonies were placed in each small tank (~ 5 cm away from each other), and thus did not come into contact with each other. These tanks were randomly designated for one of four treatments: (1) environmental temperature (~ 23 °C) and control (no-LPS); (2) environmental temperature with 5 µg ml^-1^ LPS, lyophilized from *Escherichia coli* (ATCC 25,922 strain; Chrisope Technologies, Louisiana, USA) and dissolved in sterile filtered seawater (Palmer et al., 2011); (3) elevated temperature (~ 28 °C) and control (no-LPS); (4) elevated temperature with 5 µg ml^-1^ LPS. Corals were exposed to a constant flow (from the larger aquaria) of 20 μm-filtered seawater at either environmental or elevated temperature for 2 days prior to LPS treatment. Over the LPS exposure, the system was closed by stopping the water circulation in the small tanks (raising the rim slightly above the waterline of the larger aquaria), and the seawater was carefully exchanged with relative-temperature 20 μm sterile filtered sea water with LPS (5 µg ml^-1^) or without LPS in the designated tanks. Through this method, the seawater within each small tank remained isolated, while temperatures continued to be maintained at the same values as in the large aquaria (i.e., ~ 23 °C or ~ 28 °C). The LPS treatment occurred over a period of 6 h, keeping the water moving via air stones in each small tank which flowed vigorously during the entire exposure. Once the period of LPS exposure was over, colonies were sampled from all treatment groups and immediately fixed or stored at − 30 °C for histological experiments and tissue extraction, respectively. For the whole duration of the experiment, no mortality occurred, and all coral branches appeared healthy and without any visible lesions.

### Western blotting

Western blot analysis was performed to determine the antibody specificity against selected target proteins in samples of *A. calycularis*^75–77^. Compatibility with the antibody-epitopes used was verified on the NCBI (National Center for Biotechnology Information) and UniProt databases, based on similarity between the protein sequences present in species phylogenetically close to the orange coral and those used for antibody cultivation (human TLR4, NF-kB, HSP70) (Fig. S4; Table S1). Tissues were removed from frozen samples and subsequently homogenized into polycarbonate tubes with 500 µl TBS-buffer (NaCl 150 mM, Tris–HCl 10 mM, pH 7.4) containing a complete EDTA-free cocktail of protease inhibitors (Sigma-Aldrich) on ice; the resultant slurry was then centrifuged (36,200 × g for 20 min at 4 °C). The supernatant was collected and sample absorbance was read at 595 nm (RAYTO RT-2100C) with TBS as blank, and a calibration curve defined through bovine serum albumin was used to obtain the protein concentration^78^. Coral extracts were adjusted to 0.5 mg/ml prior to the experiments. SDS-PAGE was carried out using a 4% (stacking) and a 10% (separating) polyacrylamide gel for 50 min at 190 V using a Bio-Rad mini gel kit^79^. Coral extracts were run in parallel lines, following antibody suppliers’ recommendations. Proteins separated by SDS-PAGE were electroblotted onto a nitrocellulose membrane. The gels were prepared in blotting buffer (20 mM Tris–HCl, 192 mM glycine, 20% methanol, pH 8.8), and a semi-dry blotting bath (Bio-Rad Laboratories) was used (0.8 mA cm^2^ for 75 min). The filter membrane was soaked in blocking buffer (TBS containing 3% BSA and 1% Tween-20) and incubated overnight with the following appropriate primary antibodies diluted in blocking solution (TBS containing 0.1% BSA and 1% Tween-20): polyclonal anti-TLR4 produced in rabbit (SAB5700684, Sigma-Aldrich) (1:1000); polyclonal anti-NF-kB produced in rabbit (SAB4501989, Sigma-Aldrich) (1:1000); monoclonal, anti-HSP70 produced in mouse (SAB4200714, Sigma-Aldrich) (1:1000). Membranes were then washed with blocking buffer and incubated with the appropriate secondary antibody: goat anti-rabbit IgG-alkaline phosphatase (A3812, Sigma-Aldrich) or goat anti-mouse IgG-alkaline phosphatase (A3562, Sigma-Aldrich) (1:15,000 in washing buffer, 0.1% BSA) for 1 h. Antibody binding was detected by chromogen substrate BCIP/NBT (Sigma-Aldrich).

### Histological analysis

For histological assessment, colonies were fixed in 4% paraformaldehyde suspended in PBS-buffer solution (NaCl 137 mM, KH_2_HPO_4_ 10 mM, KH_2_HPO_4_ 2 mM, KCl 2.7 mM, pH 7.6) at 4 °C. After decalcification in 10% EDTA and dehydration in ethanol, polyps were embedded in paraffin (Bio-Optica, Italy). Histological Sects. (7 μm thick) were cut with a rotary automatic microtome (Leica Microsystems HM350S, Wetzlar, Germany), stained with Hematoxylin/Eosin (H/E) and Gomori trichrome to evaluate the morphological features of the samples. Slides were analyzed with a light microscope (Leica DM750, Wetzlar, Germany), and images were obtained using an ORMA-Eurotek MDH5 scientific camera (Milan, Italy).

### Immunohistochemistry

For the immunohistochemistry assays, dewaxed sections were incubated in a blocking buffer (PBS containing 5% BSA and 1% Tween-20) for 2 h at room temperature, and then with the same primary antibodies indicated above diluted in blocking solution (PBS containing 1% BSA and 1% Tween-20; dilutions recommended by the manufacturer: anti-TLR4 1:200; anti-NF-kB 1:200; anti-HSP70 1:200) overnight at 4 °C. All slides were washed before incubation with the secondary antibodies for 90 min at room temperature. The secondary antibodies (the same indicated above) were diluted 1:50 in PBS containing 1% BSA and 1% Tween-20 and incubated for 90 min. The sections were rinsed with the washing buffer (PBS containing 1% Tween-20) and stained with the BCIP/NBT chromogen substrate (Sigma-Aldrich). In all control experiments, primary antibodies were omitted, and sections were incubated only with the secondary antibodies. Slides were analyzed by a light microscope (Leica DM750), and images were obtained using an ORMA-Eurotek MDH5 scientific camera (Milan, Italy).

### Statistical analyses

The protein marker expression results from the Western blotting were analyzed through densitometric analysis, using the open-source software Image J^80^. Furthermore, the quantification of the immune-positive stained areas (percentage of stained cells) on 6 randomly-chosen fields (45,000 μm^2^) for each slide, considering all tissue districts of corals (i.e., from oral to aboral ends), were also carried out using Image J software^80^. One-way ANOVAs were conducted in order to test differences among experimental groups. When significant differences were found, multiple comparisons using the Tukey post-hoc test were done to highlight differences between treatments. Statistical analyses were carried out using the GraphPad software (Prism 8.0, San Diego CA, USA). All experiments were performed in triplicate, and the values used were the mean ± standard deviation (SD) resulting from three independent experiments. Differences were considered significant for *p* < 0.05.

### Supplementary Information

Supplementary Information.

## Data Availability

All data generated or analysed during this study are included in this published article (and its supplementary information files).
