# Supplementary material for: Global warming-related response after bacterial challenge in Astroides calycularis, a Mediterranean thermophilic coral
Source: Sci Rep. 2024 Apr 11;14:8495. doi: 10.1038/s41598-024-58652-0 (PMC11009343; doi:10.1038/s41598-024-58652-0)
Supplement: Supplementary file 1 — Supplementary Information. [file 41598_2024_58652_MOESM1_ESM.docx]

**Global warming-related response after bacterial challenge in *Astroides calycularis*, a Mediterranean thermophilic coral**

L. Bisanti^1,2^**,** C. La Corte^1,2^, M. Dara^1,2^, F. Bertini^1,2^, M.G. Parisi^1, 2^, R. Chemello^1,2^, M. Cammarata^1,2,*^, and D. Parrinello^1,2^

^1^Department of Earth and Marine Sciences, University of Palermo, 90128 Palermo (Italy)

^2^NBFC, National Biodiversity Future Center, 90133 Palermo (Italy)

*matteo.cammarata@unipa.it

**Supplementary figures**


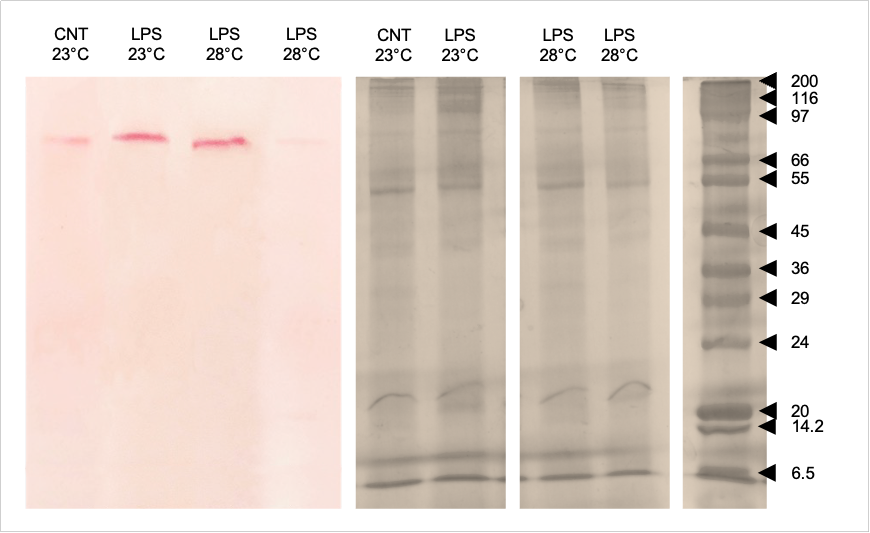


**Figure S1** Representative Western blot assay (left) and SDS-PAGE under non-reducing conditions (right) carried out on *A. calycularis* tissue extracts from the experimental treatments. Each nitrocellulose sheet was treated with anti-TLR4, the anti-rabbit IgG-alkaline phosphatase secondary antibody, and was developed with the BCIP/NBT liquid substrate system.


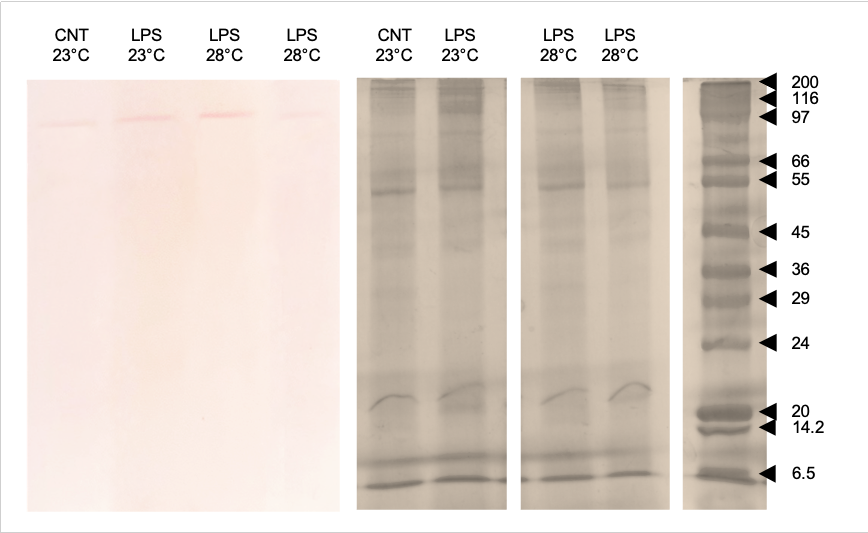


**Figure S2** Representative Western blot assay (left) and SDS-PAGE under non-reducing conditions (right) carried out on *A. calycularis* tissue extracts from the experimental treatments. Each nitrocellulose sheet was treated with anti-NF-kB, the anti-rabbit IgG-alkaline phosphatase secondary antibody, and was developed with the BCIP/NBT liquid substrate system.


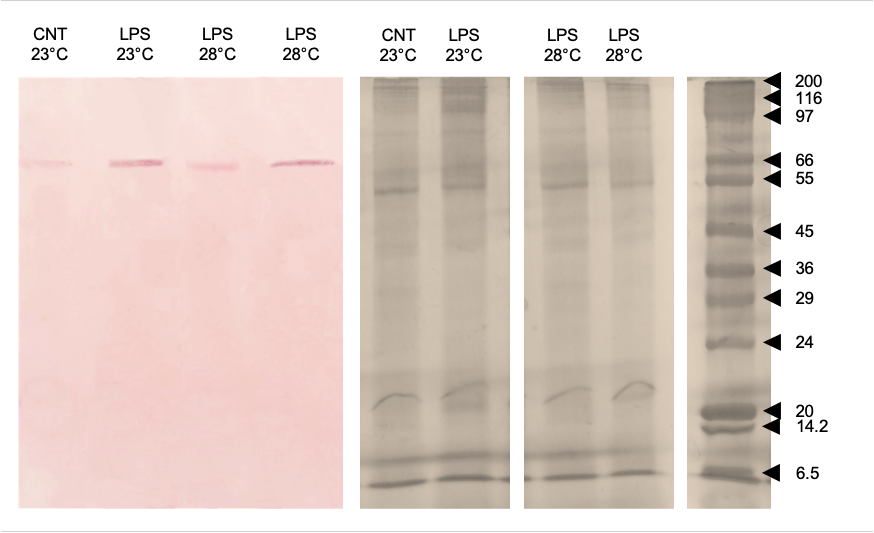


**Figure S3** Representative Western blot assay (left) and SDS-PAGE under non-reducing conditions (right) carried out on *A. calycularis* tissue extracts from the experimental treatments. Each nitrocellulose sheet was treated with anti-HSP70, the anti-mouse IgG-alkaline phosphatase secondary antibody, and was developed with the BCIP/NBT liquid substrate system.


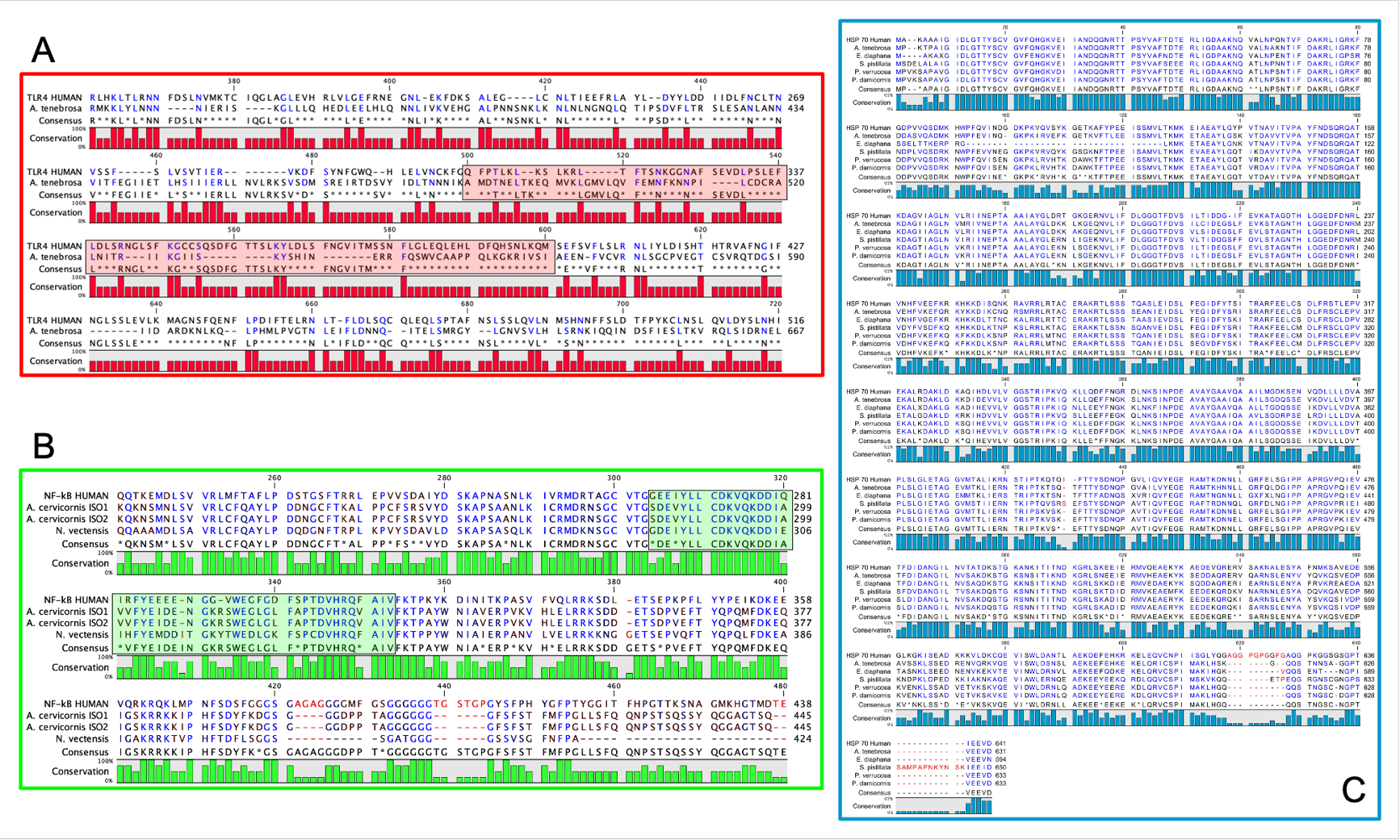


**Figure S4** The sequences of (A) TLR4, (B) NF-kB, and (C) HSP70 used for alignment with species phylogenetically close to *A. calycularis*, allowing for systematic and comprehensive identification of antigen binding sites. The consensus contained in the colored box (only for TLR4 and NF-kB; for HSP70 the entire protein is considered) improves the understanding of antigenic interactions that determine cross-reaction in the immunoblotting assays performed using *A. calycularis* tissue extracts. The references and the accession number of the sequences used in the alignment are reported in supplementary Table S1.


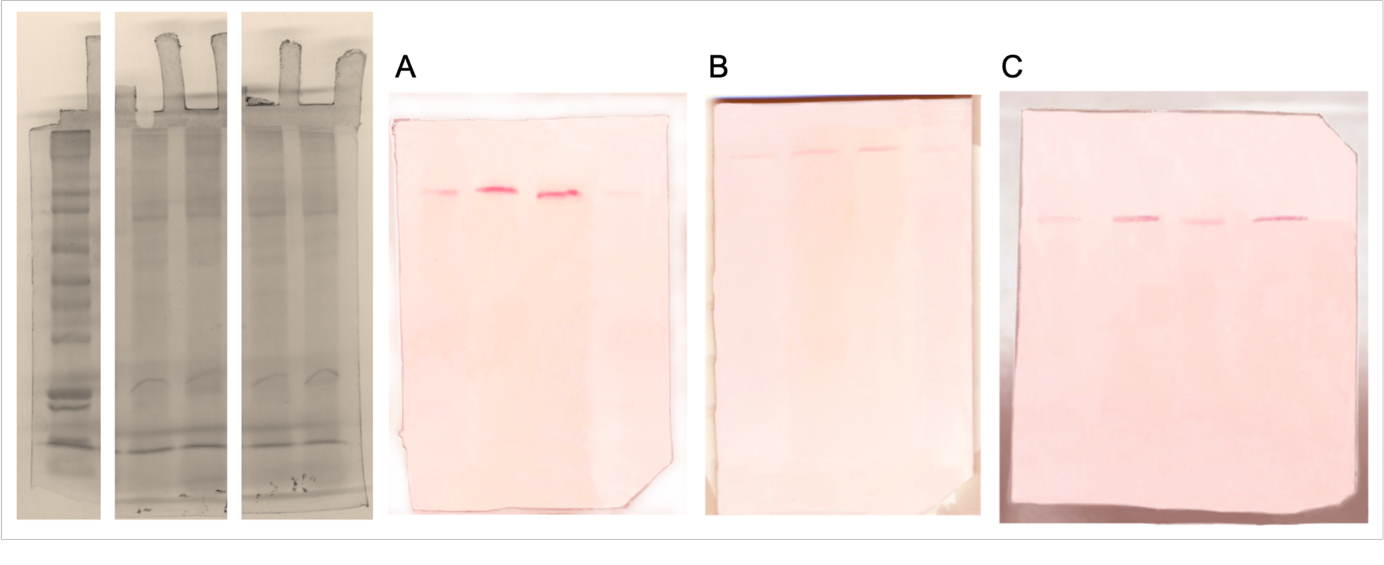


**Figure S5** Full-length representative pictures of Western blot assays and SDS-PAGE under non-reducing conditions carried out on *A. calycularis* tissue extracts from the experimental treatments. Each nitrocellulose sheet was treated with anti-TLR4 (A), -NFkB (B) and -HSP70 (C), the anti-mouse IgG-alkaline phosphatase secondary antibody, and was developed with the BCIP/NBT liquid substrate system.

**Supplementary tables**

| **Species** | **Protein** | **Accession number** |
| --- | --- | --- |
| *Actinia tenebrosa* | Toll-like Receptor (TLR) | GenBank: ALG40986.1 |
| *Homo sapiens* | Toll-like Receptor 4 (TLR4) | UniProtKB O00206 |
| *Nematostella vectensis* | Nuclear Factor kappa B (NF-kB) | GenBank ADQ57372.1 |
| *Acropora cervicornis* | Nuclear Factor kappa B (NF-kB) ISO1 | GenBank UCR60993.1 |
| *Acropora cervicornis* | Nuclear Factor kappa B (NF-kB) ISO2 | GenBank UCR60992.1 |
| *Homo sapiens* | Nuclear Factor kappa B (NF-kB) | UniProtKB P19838 |
| *Actinia tenebrosa* | Heat Shock Protein 70 (HSP70) | GenBank XP_031562268.1 |
| *Aiptasia diaphana* | Heat Shock Protein 70 (HSP70) | GenBank XP_028514728.1 |
| *Stylophora pistillata* | Heat Shock Protein 70 (HSP70) | GenBank AKC91104.1 |
| *Pocillopora verrucosa* | Heat Shock Protein 70 (HSP70) | GenBank QDO73499.1 |
| *Pocillopora damicornis* | Heat Shock Protein 70 (HSP70) | GenBank BAD89541.1 |
| *Homo sapiens* | Heat Shock Protein 70 (HSP70) | UniProtKB P0DMV8 |

**Table S1** References and accession number of the sequences used for the sequence alignment of species phylogenetically close to the orange coral *A. calycularis* with the antigenic epitopes of the antibodies employed in the immunoblotting assay.
